# Soft Composites Filled with Iron Oxide and Graphite Nanoplatelets under Static and Cyclic Strain for Different Industrial Applications

**DOI:** 10.3390/polym14122393

**Published:** 2022-06-13

**Authors:** Vineet Kumar, Md Najib Alam, Sang Shin Park

**Affiliations:** School of Mechanical Engineering, Yeungnam University, 280, Daehak-ro, Gyeongsan 38541, Korea; vineetfri@gmail.com (V.K.); mdnajib.alam3@gmail.com (M.N.A.)

**Keywords:** graphite nanoplatelets, iron oxide, silicone rubber, composites, energy harvesting, magnetic sensitivity

## Abstract

Simultaneously exhibiting both a magnetic response and piezoelectric energy harvesting in magneto-rheological elastomers (MREs) is a win–win situation in a soft (hardness below 65) composite-based device. In the present work, composites based on iron oxide (Fe_2_O_3_) were prepared and exhibited a magnetic response; other composites based on the electrically conductive reinforcing nanofiller, graphite nanoplatelets (GNP), were also prepared and exhibited energy generation. A piezoelectric energy-harvesting device based on composites exhibited an impressive voltage of ~10 V and demonstrated a high durability of 0.5 million cycles. These nanofillers were added in room temperature vulcanized silicone rubber (RTV-SR) and their magnetic response and piezoelectric energy generation were studied both in single and hybrid form. The hybrid composite consisted of 10 per hundred parts of rubber (phr) of Fe_2_O_3_ and 10 phr of GNP. The experimental data show that the compressive modulus of the composites was 1.71 MPa (virgin), 2.73 (GNP), 2.65 MPa (Fe_2_O_3_), and 3.54 MPa (hybrid). Similarly, the fracture strain of the composites was 89% (virgin), 109% (GNP), 105% (Fe_2_O_3_), 133% (hybrid). Moreover, cyclic multi-hysteresis tests show that the hybrid composites exhibiting higher mechanical properties had the shortcoming of showing higher dissipation losses. In the end, this work demonstrates a rubber composite that provides an energy-harvesting device with an impressive voltage, high durability, and MREs with high magnetic sensitivity.

## 1. Introduction

A polymer composite containing an electrical-conducting reinforcing filler showing piezoelectric behavior, in a hybrid with iron particles, and which exhibits a magnetic response, is an interesting topic in research. Various new routes are under investigation to meet an increase in energy demands. The use of polymer composites in piezoelectric energy-harvesting-based devices tends to produce few volts via mechanical motion [1,2]. On the other hand, iron particles in a polymer composite are categorized as magneto-rheological elastomers (MREs) and their mechanical properties are sensitive to a magnetic field. Polymer composites with hybrid fillers containing iron particles and electrically conductive reinforcing fillers received great attention [3,4]. Research and review studies by Zaghloul et al. reveals fatigue and wear properties of polymer composites for various useful applications [5,6,7].

MREs are well known and have been investigated for many decades [8]. MREs contain magnetic particles that tend to orient in a magnetic field, thereby influencing mechanical properties such as the modulus. Various types of polymer matrix were used in designing MREs. These polymer matrixes are elastomers, such as natural rubber [9], butadiene rubber [10], or silicone rubber [11]. Among them, silicone rubber is frequently used, as it is easily processed and cured and has low hardness, all of which make it a candidate for soft composites [12]. It is well known that silicone rubber can be categorized on the basis of vulcanization type. These are high temperature vulcanized (HTV) and room temperature vulcanized (RTV) silicone rubber [13]. Room temperature vulcanized silicone rubber can be used in various applications, such as insulating coatings, strain sensors or actuators, and piezoelectric energy harvesting [14].

Recently, carbon-based secondary fillers, such as MWCNTs were added to MREs containing iron particles to improve the mechanical properties of MREs [15]. These secondary reinforcing fillers form synergy with the iron particles and produce high performance MREs [16]. The type of carbon-based secondary nanofiller used in MREs also influences their damping properties. MWCNTs are one-dimensional in nature and have a tube-shaped morphology and high aspect ratio that significantly improves mechanical and electrical properties; they act as a catalyst to properties of MREs in terms of energy harvesting [17]. Another way to produce high performance MREs is to produce them with iron particles characterized by small particle size, high surface area, high aspect ratio, and favorable morphology so that they orient easily in a magnetic field [18]. In addition to the type of iron particle, and the type of secondary reinforcing filler, the type of polymer matrix has a significant role in affecting properties of MREs as detailed above [19].

In this work, soft silicone rubber (hardness below 65) composites are used to produce high performance MREs which exhibit an improved magnetic response. The type of iron particle is Fe_2_O_3_ and type of secondary reinforcing nanofiller is GNP. MREs with different features were demonstrated as (a) MREs with iron particles only, which only show a magnetic response; and (b) Specimens with GNP only, which only exhibit piezoelectric energy harvesting. To the best of the authors’ knowledge, the MREs exhibiting both a magnetic response and piezoelectric energy harvesting has not been fully understood and is, most recently, a hot topic of research and development in MREs. In previous work, Mannikkavel et al. show a robust piezoelectric energy-harvesting device based on a composite made from HTV–RTV silicone rubber with an MWCNT as the electrode. The voltage generation was, however, lower and was approximately 1 V [20]. In this work, with the addition of a hybrid filler in the substrate, the voltage increases to as high as 10 V and durability was enhanced to 0.5 million cycles. This work is, thus, advantageous in terms of voltage and durability.

## 2. Materials and Methods

### 2.1. Materials

The MREs were fabricated with room temperature vulcanized silicone rubber (RTV-SR, obtained from Shin-Etsu, Tokyo, Japan) as the elastomeric matrix (commercial name KE-441-KT, obtained from Shin-Etsu, Tokyo, Japan) which was transparent in appearance. The iron oxide (Fe_2_O_3_) and graphite nanoplatelets (GNP) were used as nanofillers. The Fe_2_O_3_ had a surface area of around 50 m^2^/g, and particle size below 50 nm and was used as iron particles in MREs fabricated in this work; it was obtained from Alfa-Aesar, Ward Hill, Massachusetts, USA. The GNP, on the other hand, had a surface area of around 125 m^2^/g, thickness of 4–5 nm and lateral size of ~2 µm. Both nanofillers (Fe_2_O_3_ and GNP) were used in single and hybrid forms in the MREs and their improved properties are demonstrated. The vulcanizing agent was “CAT-RM”—a whitish-blue liquid—used for curing and obtained from Shin-Etsu, Tokyo, Japan. The mold-releasing agent spray was white liquid powder, obtained from Nabakem, Gyeonggi-do, Korea.

### 2.2. Preparation of Composites

The MREs and piezoelectric energy-harvesting-based composites were prepared by the solution-mixing technique and the procedure is reported elsewhere [21]. The preparation of composites was initiated by spraying the molds with mold-releasing agent. The molds were sprayed and left for 1–2 h before use for composite sample preparation. An amount of 100 phr of RTV-SR solution (liquid state of rubber without any solvent) was then mixed with different concentrations of nanofillers, both in single and hybrid forms, as detailed in Table 1. The filler–rubber mixing lasted for 10 min. Next, 2 phr of vulcanizing agent was added and mixed for 1 min. The composite was poured into the molds and kept at room temperature for vulcanization for 24 h before removal for testing.

### 2.3. Characterization Technique

The morphology of Fe_2_O_3_ and GNP were studied by SEM microscopy (S-4800, Hitachi, Tokyo, Japan). All samples were coated with platinum before the SEM micrographs were taken. The coating was performed to facilitate the surface conduction for the SEM measurements. The SEM was also used to investigate filler dispersion. For these tests, the cylindrical sample (20 × 10 mm) was sectioned by surgical blade into thin slices of 0.5 mm-thick samples. The applied voltage for SEM measurements was 10 kV and the working distance was ~11 mm. The compressive cyclic and static mechanical measurements were performed by a universal testing machine (UTS, Lloyd Instruments, West Sussex, UK). The cylindrical samples were used in measurements of compressive tests at a strain rate of 2 mm/min.

**Figure 1:**
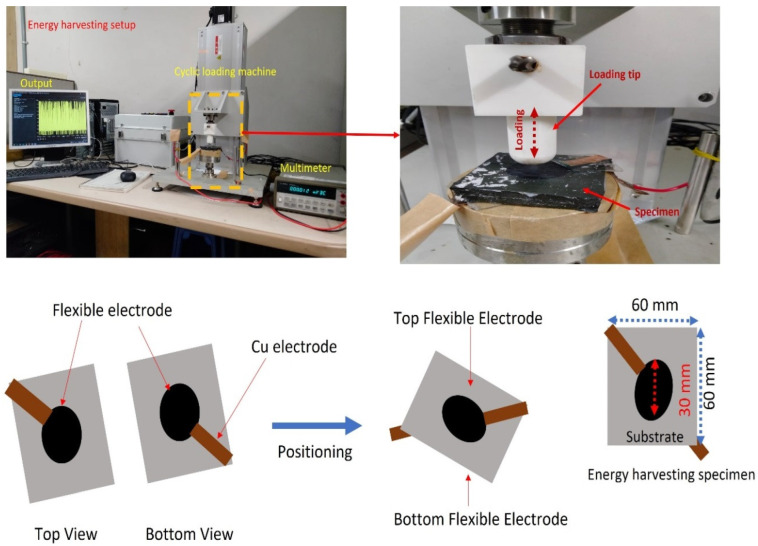
Piezoelectric energy-harvesting set-up with details on device dimensions and their assembly.

The compressive strain applied for static and cyclic tests was 0–35% max and 30% max, respectively, for 100 cycles. The tensile mechanical measurements were taken at 100 mm/min on dumbbell shaped samples with a gauge length of 25 mm and thickness of 2 mm using the UTS machine (Lloyd instruments, West Sussex, UK). The mechanical properties were tested according to DIN 53 504 standards. The optical images of the set-up for compressive and tensile measurements are reported elsewhere [21]. Hardness tests were taken on cylindrical samples using a Westop durometer, according to ASTM D 2583 standards. The magnetic response measurements and stress–relaxation tests were performed on cylindrical tests using the UTS machine and the optical image of the set-up is displayed elsewhere [22]. The number of samples tested for mechanical properties were 3 for each tensile/compressive/hardness test. For the compressive and hardness tests, the sample was manually gripped, while a pneumatic grip was used for tensile measurements. The magnetic response of the MREs was performed at 90 mT and the stress–relaxation was performed under on–off of the magnetic field. The piezoelectric energy harvesting was performed using a mechanical testing machine (Samick-THK, Daegu, Korea) under cyclic strain and the optical images of the set-up, optical image of the sample, dimension of electrode and thickness of sample under compressive strain are detailed in Figure 1 above.

## 3. Results

### 3.1. Morphology of Fe_2_O_3_ and GNPs as Filler Particles

**Figure 2:**
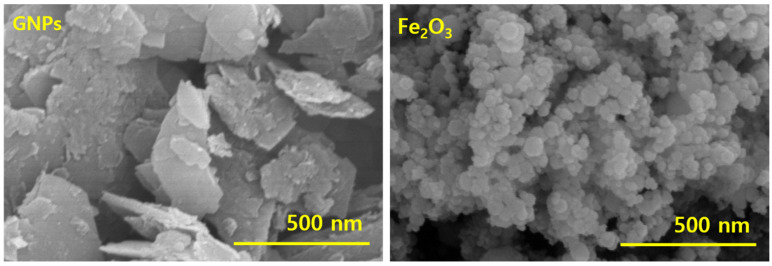
SEM showing GNPs and Fe_2_O_3_.

The morphology of fillers is well known to affect the properties of the composites. Fillers with favorable morphology are easily dispersed in the polymer matrix and their uniform dispersion simulates the properties [23]. Here, two types of nanofillers with different morphology were used (Figure 2) and their use as MREs and energy harvesters was studied. Fe_2_O_3_ with oval 0-Dimensional (0D) morphology was used as iron particles to make the composite magnetically active both in single and hybrid forms and their improved properties were studied. GNP with sheet-like morphology with 3D structure forms, 3D filler networks, and their synergy with Fe_2_O_3,_ was investigated. The single and hybrid forms of GNPs create electrically conductive networks which are useful for energy harvesting. Briefly, MREs were prepared using Fe_2_O_3_ as iron particles and GNPs as secondary reinforcing and conductive fillers to make the MREs useful for energy harvesting.

### 3.2. Filler Dispersion of Composites

#### 3.2.1. Through SEM Microscope

**Figure 3:**
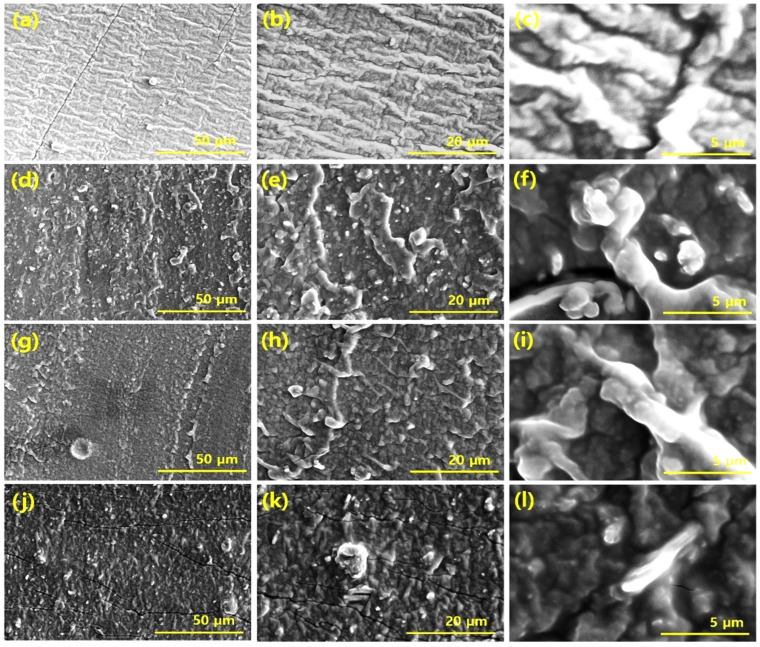
SEM micrographs of different composites; (**a**–**c**) virgin, (**d**–**f**) GNP composites, (**g**–**i**) Fe_2_O_3_ composites, (**j**–**l**) hybrid composite.

Filler dispersion plays an important role in influencing the properties of composites. A composite in which the filler dispersion is uniform tends to show better properties than those with poor filler dispersion [23]. Here, we employed the SEM technique to determine filler dispersion in composites. A number of SEM images were studied and their representative images per sample are shown in Figure 3. The filled composite SEM images show that the filler particles form long-range networks throughout the silicone rubber matrix. Moreover, uniform filler dispersion was noticed in the SEM images. The SEM images of composites based on both Fe_2_O_3_ and GNP, as the only filler, shows improved dispersion, and evidence of filler aggregation was absent. Moreover, the composites based on hybrid fillers tend to show synergy between the Fe_2_O_3_ and GNP particles and, in a few cases, evidence of exfoliation of GNP particles was noticed. In some cases, Fe_2_O_3_ particles were found in the vicinity of the GNP particles and in a few cases GNP platelets were found in the vicinity of bunches of Fe_2_O_3_ particles. The GNP platelets were held together by van der Waals forces which are weak and easily exfoliated against mechanical strain, such as during mixing and under mechanical compressive or tensile strain, and leads to distribution of the exfoliated GNP particles [24]. On the other hand, the Fe_2_O_3_ nanoparticles with a particle size below 50 nm were also distributed uniformly due to nano-dimensions and their favorable oval morphology that allows easy dispersal in the composite, both in single and hybrid states. Moreover, the exfoliated GNP particles help to prevent aggregation of Fe_2_O_3_ particles, thereby leading to their uniform dispersion as observed in the SEM images.

#### 3.2.2. Through Elemental Mapping

**Figure 4:**
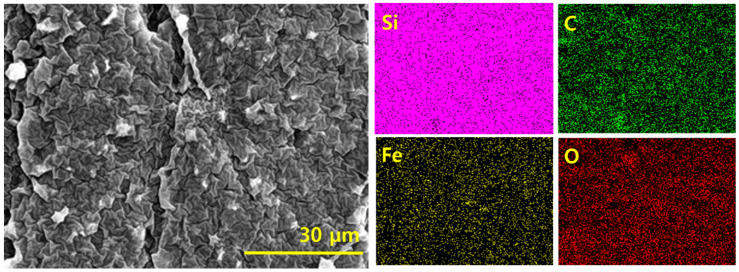
SEM and element maps of different elements in hybrid composite.

The filler dispersion can be further assessed with the help of elemental mapping [25]. In the present hybrid composite, four types of elements were noticed, specifically, Si from silicone rubber, C from GNP, and Fe and O from Fe_2_O_3_ (Figure 4). All the maps show that the elements are densely distributed, whether Si from silicone rubber or C, Fe, or O from different nanofillers present in the hybrid composite. The element maps further justify the absence of aggregation of nanofillers in the composite. The SEMs in Figure 2 and element maps in Figure 3 agree with each other.

### 3.3. Mechanical Properties

There are three stages of filler network formation in rubber composites. Firstly, at the low filler volume fraction of the filler, filler network formation is initiated and the modulus increases linearly up to a certain amount of filler. The modulus then increases exponentially at a certain amount of filler volume fraction. This is the stage of the filler percolation threshold in which long-range and continuous filler networks are formed [26,27]. The third stage is the filler aggregation stage in which the modulus falls and results in the presence of excess filler particles in the composite. In this work, an amount of filler was added at the second stage; i.e., at the filler percolation threshold at which exponentially high properties are witnessed. Beside the amount of filler, the type of strain also affects the mechanical properties of the composites. In this work, compressive static and cyclic strains and tensile strain are applied, and the behavior of mechanical properties are studied and presented below.

#### 3.3.1. Under Static Compressive Strain

**Figure 5:**
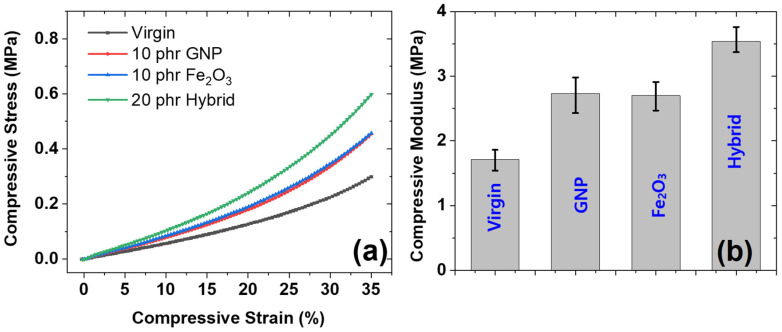
Compressive mechanical properties; (**a**) compressive stress–strain of the composites, (**b**) compressive modulus of different composites.

The mechanical behavior under static compressive strain was studied and is presented in Figure 5. Figure 5a shows the compressive stress–strain for different composites prepared in this work. It was found that the stress increases with increasing compressive strain. It is also interesting to note that the compressive stress was linear up to 15% of compressive strain and then increases exponentially. Such a behavior is based on filler networks and the packing fraction of polymer chains and filler particles [28]. It is also interesting to note that as the volume fraction of the filler particles increased to 20 phr (hybrid composite), the mechanical properties increased at all strains. The mechanical properties were higher for the hybrid composite than for GNP and Fe_2_O_3_ as the sole filler in composites. In addition, the GNP as the sole filler shows higher reinforcement than Fe_2_O_3_ as the sole filler; this is due to the higher reinforcement exhibited by GNP in rubber composite [29]. On the other hand, Fe_2_O_3_ tends to exhibit lower reinforcing properties due to its poor reinforcing effect in composites.

The behavior of compressive modulus was studied and is presented in Figure 5b. It was found that the reinforcing effect was higher for GNP-filled composites and highest for hybrid-filled composites. The higher mechanical compressive modulus for GNP is due to the higher reinforcing effect of GNP particles due to their favorable sheet-like morphology that allows easy dispersal and because they exhibit a higher compressive modulus [30]. Beside this, the hybrid-filled composites exhibit the highest compressive modulus; this is due to presence of the higher amount of filler (20 phr) in the hybrid composite than when GNP and Fe_2_O_3_ are used as single fillers (10 phr each). The higher filler content leads to higher reinforcements and, thus, higher mechanical properties.

#### 3.3.2. Under Cyclic Compressive Strain

**Figure 6:**
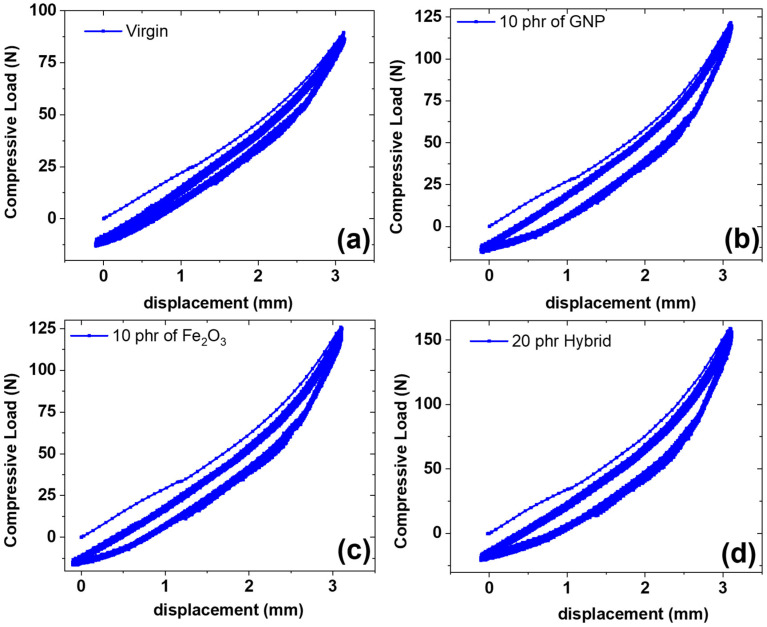
Compressive cyclic mechanical properties; (**a**) virgin composite, (**b**) GNP composite, (**c**) Fe_2_O_3_ composite, (**d**) hybrid composite.

Figure 6 shows multi-hysteresis measurements for different types of composites. The measurements are useful for determining the heat dissipation under cyclic strain. It was found from the tests that the dissipation losses were lower for virgin and Fe_2_O_3_ composite samples, higher for the GNP composite, and highest for the hybrid composite. The lower dissipation losses for virgin samples are due to the absence of filler particles and, therefore, lower hysteresis losses [31]. In the case of Fe_2_O_3_, the dissipation was also lower. This was attributed to the lower reinforcing effect of the Fe_2_O_3_ filler and, therefore, lower hysteresis losses. The higher hysteresis losses for the GNP and hybrid composites are due to the higher reinforcing effect of GNP in both GNP as a single filler and a hybrid filler [32].

The highest dissipation losses in the hybrid composite, which exhibits higher reinforcement, are due to the presence of higher filler content, as described in the mechanical properties’ sections. Thus, although the hybrid exhibits higher mechanical properties, it shows the limitation of exhibiting higher dissipation losses, which is a drawback. It is also interesting to note that the first hysteresis cycle exhibits higher dissipation losses in all composites and stabilizes after subsequent cycles [33]. This can be attributed to the break-down of new bonds under strain for the first cycle and the formation of new bonds once the stress is removed. The stabilization of hysteresis losses in subsequent cycles is due to the attainment of equilibrium between breakdown and formation of bonds in the filler networks, as justified in Figure 6. Our future work will involve the functionalization of the filler to enhance polymer-filler interactions, thereby improving stress transfer from the polymer matrix to filler particles to suppress dissipation losses, which will be an advantage for the industrial application of composites.

#### 3.3.3. Under Static Tensile Strain

**Figure 7:**
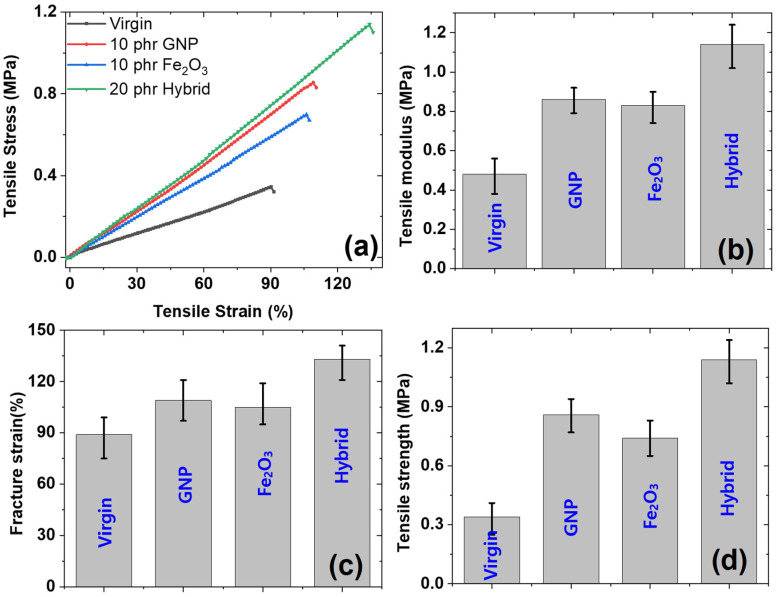
Tensile mechanical properties; (**a**) profiles of stress–strain of different composites, (**b**) tensile modulus for different composites, (**c**) fracture strain of different composites, (**d**) tensile strength of the composites.

The behavior of the mechanical properties under tensile strain until fracture was investigated. Properties, specifically, the tensile modulus, tensile strength, and fracture strain of the composites, were determined (Figure 7). It was found that the tensile stress increases linearly with increasing tensile strain until fracture strain (Figure 7a). It was also interesting to note that the tensile mechanical properties were higher for the GNP and hybrid composites and lower for the Fe_2_O_3_ and virgin composites. A similar trend was found in the compressive mechanical properties. Thus, our experimental data are consistent with each other. The higher tensile properties for GNP and hybrid composites are due to the higher reinforcing ability of GNP in both single and hybrid forms [34]. The highest mechanical properties of the hybrid are due to its higher filler content (20 phr) compared with GNP and Fe_2_O_3_ as single fillers (10 phr).

The tensile modulus (Figure 7b), fracture strain (Figure 7c) and tensile strength (Figure 7d) were studied for different composites. The properties were lowest for the virgin and Fe_2_O_3_-filled composites, while they were higher for the GNP and hybrid composites. The higher tensile modulus for the GNP and hybrid-filled composites is due to the induced stiffness of GNP for a rubber matrix and the high reinforcing property of GNP, as detailed in the literature [35]. The higher fracture strain and tensile strength of the GNP and hybrid composites are due to the lubricating nature of GNP, in which the graphene planes stack together via van der Waals forces that tend to repel each other, leading to higher fracture strain and tensile strength [36]. It is also interesting to note that hybrid composites exhibit the highest mechanical properties, and a sort of synergism was noticed for composites, especially in fracture strain and tensile strength. The higher properties of the hybrid composites are also due to the higher filler content (20 phr) compared with other-filled composites, which cause higher reinforcements for rubber matrix.

### 3.4. Hardness of Rubber Composites

**Figure 8:**
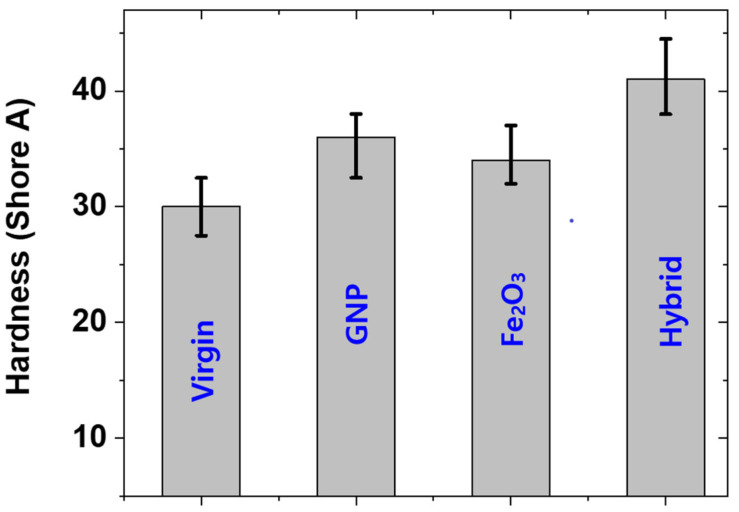
Hardness of different composites.

The hardness of composites is useful for determining whether a composite is soft or not. Generally, a composite with a hardness below 65 is termed a soft composite [25]. In this work, hardness was determined. It was found that the hardness was well below 65 (Figure 8). This means that the composites prepared in this work are soft and useful for various applications, such as soft robotics etc. It was also interesting to note that the behavior of hardness is in agreement with the behavior of mechanical properties, for example, the modulus of the composites and hardness are in agreement with each other. Moreover, the hardness of GNP was higher than that of Fe_2_O_3_ as the sole filler and the hardness was highest for the hybrid composite. The higher hardness of GNP is due to the higher reinforcing capacity of GNP and the highest hardness of the hybrid composite is due to the high filler content (20 phr) compared with other fillers.

### 3.5. Industrial Applications

#### 3.5.1. Piezoelectric Energy-Harvesting Tests

**Figure 9:**
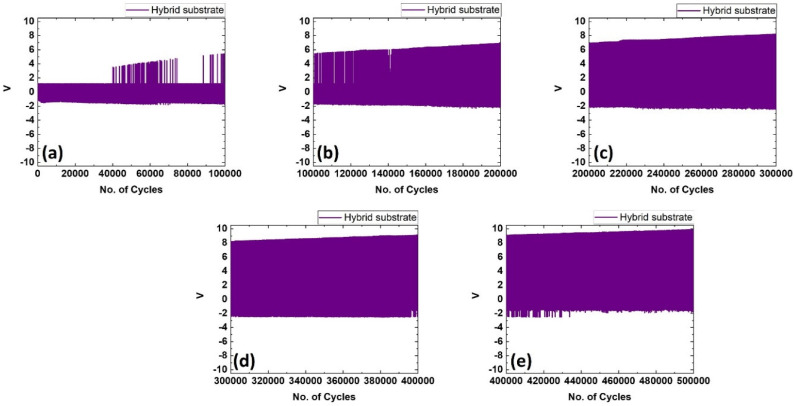
Piezoelectric energy-harvesting device cycles (**a**–**e**) output-voltages for number of cycles from 0 to 0.5 million.

In this work the substrate was made of reinforcing GNP+Fe_2_O_3_ hybrid nanofillers with RTV-SR. The electrode was made of 2 phr of MWCNT+ 2 phr of MoS_2_ reinforced with RTV-SR. The flexible energy-harvester substrate thickness was 8 mm and a 0.2 mm-thick electrode was painted on both the sides. The load was applied for a displacement of 4 mm, with the hemispherical loader having a diameter of 21 mm. A constant amplitude of displacement was provided for the entire cyclic loading. During the starting cycles, the obtained voltage value was higher than 1.5 V. This value was constant for up to 40,000 cycles (Figure 9). This was due to the energy of charge carriers present in the dielectric material [37]. After 40,000 cycles, the voltage value started to enhance at the higher rate. This was because of the geometry of the specimen. The specimen construction made it a capacitor. The charging effect produced in the electrode was due to the continuous voltage production because of repeated loading. Once sufficient charging occurs, on further loading, the capacitive geometry specimen is able to multiply the voltage production. When the applied load is continuous, the saturation in the charging of the electrode occurs and a regular voltage output is obtained from the specimen [38]. Uneven voltage output results in the variation in the activation of charge carriers during loading of the specimen [39]. During 100,000 cycles of loading, the voltage output was above 5 V. This value steadily increased with a further increase in the number of cycles. There was a sharp increase during 100,000 to 200,000 cycles. The value of voltage output during 200,000 cycles was around 7 V. In the 200,000 to 300,000 phase, the voltage value steadily increased. This is due to the energy of charge carriers occurring as the rate increases. In the course of 300,000 cycles, the maximum voltage value of above 8 V obtained. From 300,000 to 400,000 cycles, the voltage value increased again. The voltage value of 9 V was obtained by the specimen during the repeated loading of 400,000 cycles. During 400,000 to 500,000 cycles, the voltage value increased slightly. The maximum obtained voltage value of around 10 V was achieved during 500,000 cycles. In the curve, the top portion is the voltage output due to loading of the specimen. The bottom portion of the voltage formation is due to the free movement of the specimen. The forced loading was able to achieve a higher voltage output than the free movement of the specimen. The MWCNT present in the electrode has the property of achieving higher conductivity for the electrode, which can collect the charge formation during loading. MoS_2_ has a lubricating nature and is able to achieve higher fracture toughness when combined with the silicone rubber. This property reduces crack formation on the electrode during repeated loading. The reinforcement present in the substrate has an effect on the dielectric property. The nano reinforcements in the electrode and substrate collectively enhance the voltage output.

#### 3.5.2. Magnetic Response Tests of MREs

**Figure 10:**
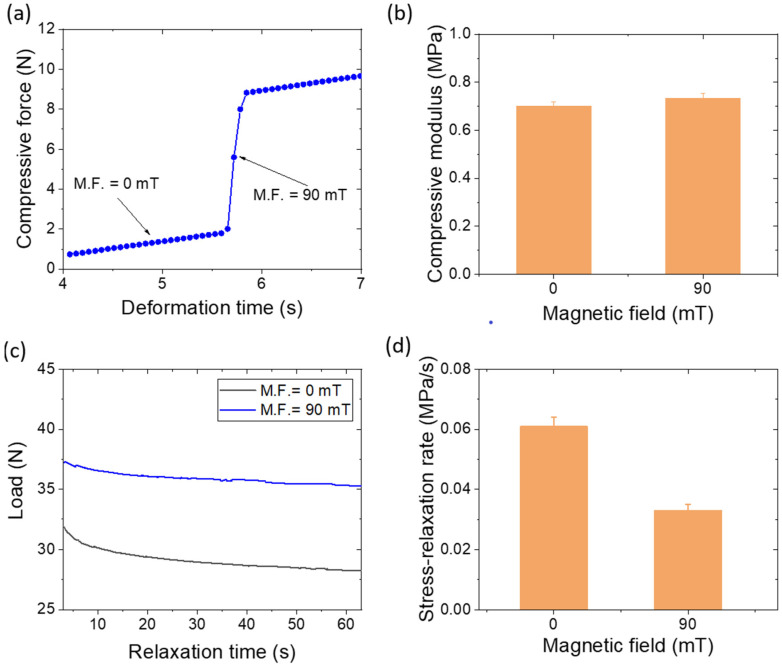
Magneto-mechanical properties of 40 phr Fe_2_O_3_-filled silicone rubber composite; (**a**) compressive force under a magnetic cycle, (**b**) magnetic effect on modulus, (**c**) load relaxation curves within 3–63 s with 10% deformation of cylindrical sample, and (**d**) magnetic effect on the stress–relaxation rate.

The above composites with lower Fe_2_O_3_ can sense the external magnetic field but it was not significant in the change of stiffness of the rubber composites. To study the magneto-mechanical response properties at a lower external magnetic field (90 mT), a higher amount of 40 phr Fe_2_O_3_ was utilized. Figure 10a indicates the compressive mechanical profile under one cycle of external magnetic field described elsewhere [38,39]. The lowest slope in the compressive profile is due to isotropic filler distribution [38,39], whereas the highest slope is due to a fast change in the force value because of the attraction of induced magnetic poles [38,39] and is regarded as a response force. The medium slope is regarded as the true value for the anisotropic filler orientation in the presence of an externally induced magnetic field. The response force without a sample was 7.5 N in our experimental system. A slight decrease in the response force was observed because some amount of force is utilized to orient the filler particles in the direction of the external magnetic field. After the complete orientation of filler particles, the composite shows anisotropic behavior with a higher mechanical modulus. Figure 10b shows the changes in the mechanical modulus from isotropic to anisotropic filler distribution. A greater change in the modulus indicates that the composites can be utilized as magnetically derived flexible actuators in many smart applications [40,41].

A stress–relaxation experiment was also undertaken to observe the filler–filler interactions in the presence of an externally applied magnetic field [42,43]. Viscoelastic materials such as rubber undergo stress relaxation due to the strain-generated structures in the materials. Depending upon the direction of the applied magnetic field, the stress–relaxation rate can be increased or decreased. In our experimental condition, we found a decrease in the stress–relaxation rate because the flow of the matrix and the applied magnetic field acted in the reverse direction. The load relaxation curves in the absence and presence of 90 mT external magnetic fields are provided in Figure 10c. A significant reduction in the stress–relaxation rate was observed in the presence of the external magnetic field (Figure 10d), which was due to induced filler–filler interactions [42,43] which can restrict the movement of polymer chains and improve the stiffness of the composite.

## 4. Conclusions

The composites prepared in this work exhibit magnetic sensitivity and act as an energy-harvesting device. The piezoelectric energy-harvesting device generates an impressive voltage of around 10 V, high stability, and durability of more than 0.5 million cycles. From the experimental measurements, the virgin and Fe_2_O_3_-filled composites show poor mechanical properties, while the GNP and hybrid-filled composites show higher mechanical properties. However, multi-hysteresis measurements show that GNP and hybrid composites exhibit higher dissipation losses, which is a disadvantage of using these composites for industrial applications. The stress-transfer from the polymer matrix to filler particles can, however, be enhanced by improving interfacial interaction. Our future work will address the improvement of interfacial interaction, thereby suppressing the dissipation losses to make the composites more promising.

In conclusion, this work provides the readers with a method of obtaining MREs which show magnetic sensitivity and an impressive energy-generating device with high durability which is an interesting current research. This study also highlights that RTV-SR is suitable for making MREs and stretchable flexible devices. The work demonstrates that with the addition of the hybrid filler in a substrate, the voltage is enhanced as high as 10 V and durability greatly improved to as high as 0.5 million cycles. Moreover, the RTV-SR and composites prepared from this rubber were soft, with a hardness below 65, and suitable for soft applications, such as flexible electronics. In conclusion, this work recommends that hybrid filler must be used to obtain higher mechanical properties even though obtaining higher dissipation losses is a demerit. Nevertheless, the substrate with the hybrid filler exhibits higher energy generation and shows a prolonged durability, as demonstrated in Figure 9.

## Figures and Tables

**Table 1:** Formulation table of the RTV-SR based composites.

Formulation *	RTV-SR (Phr)	Fe_2_O_3_ (Phr)	GNP (Phr)	Vulcanizing Solution (Phr)
Virgin	100	-	-	2
Fe_2_O_3_ only	100	10	-	2
GNP only	100	-	10	2
Hybrid	100	10	10	2

* 10 phr of GNP and 10 phr of Fe_2_O_3_ were selected because these concentrations are filler percolation of these fillers and dominant effect of filler in reinforcing composites can be witnessed.

## Data Availability

The data presented in this study are available on request from the corresponding author.
